# A Practical Treatise on Diseases of the Joints

**Published:** 1834-07-01

**Authors:** 


### A PRACTICAL TREATISE ON DISEASES OF THE JOINTS. By *W. J. Wickham*, Surgeon to the County Hospital, Winchester. 8vo. pp. 178. Winchester, 1833.

MR. WICKHAM, an industrious and an able provincial surgeon, informs us in his preface that he has devoted his attention to the investigation of the diseases of the joints for the period of sixteen years. He has enjoyed extensive opportunities of observation in the county hospital to which he is attached. He acquaints us also that he has made little use of the works of former writers, and trusted, "perhaps too much," to his personal sources of information.

The plan of Mr. Wickham is to treat, in order, of the morbid affections of the various tissues which enter into the composition of the joints. He considers, seriatim, disease of the bony structure—of the cartilage—of the synovial membrane—of the cellular tissue—of the ligaments. He concludes by a notice of the general terminations of articular disease, and by a separate chapter on disease of the hip-joint.

We shall endeavour to glean from the work before us those portions which derive a particular interest from the personal observation or experience of the author. So much of the descriptions is necessarily indistinguishable from that of other and preceding writers, that we should not be justified in offering a complete and consecutive analysis.

Mr. Wickham commences his account of the diseases of the bony structures of the joints by remarks on exostosis.

He adopts the division proposed by Sir Astley Cooper in his Surgical Essays, into the periosteal and the medullary exostosis. The former he seems to consider as in all cases a simple growth, the latter as usually malignant. We feel disposed to doubt the justice of this absolute distinction, for reasons which we shall state after laying before our readers the opinions of Mr. Wickham. He observes with respect to periosteal exostosis, that—

"The bony tumour, or exostosis properly so called, which has a circumscribed origin from beneath the periosteum, is of rare occurrence within the capsular ligament; such tumours more frequently form externally to the joint, and proceed, by their excessive and inconvenient growth, to impede its motions.

I have seen small exostoses on the patella, also on the epiphysis of the tibia.

On the humerus and fibula I have seen them on dissection; close upon the shoulder-joint in the one case, and on the ankle in the other, and projecting towards the articulations, so as to cause considerable inconvenience.

A young woman underwent the removal of an exostosis by my friend and colleague Mr. H. Lyford; its size and shape were that of the little finger. Its origin was just without the capsular ligament of the shoulder-joint, on the fore and inner part of the humerus; the direction of its point was upwards and inwards, towards the clavicle and sternum. It had already extended far enough to impede the motion of the joint in some degree. The action of the pectoralis was thereby somewhat hindered, so that when the arm was attempted to be brought inwards by that muscle, it locked at about half its action.

The operation was easy, the tumour breaking off under the pressure of the hand. The recovery of the motion of the joint was complete. The nature of the tumour was that of the periosteal exostosis of Sir Astley Cooper. Otherwise than by its inconvenient position, the character of the tumour was perfectly innocent.

The bone spavin, which is a frequent cause of lameness in the horse, answers to the description of the periosteal exostosis.

I have never known this species of disease lead on to the destruction of the other textures of the joint." 8.

Mr. Wickham refers to an extraordinary instance of spiculated exostosis related in the 41st volume of the Philosophical Transactions. Every joint, with the exception of the wrists and knees, was anchylosed by the osseous productions extending from one bone to another.

Mr. W. has ornamented his work with a copy of the original drawing of this curious case. Perhaps it is more adapted to excite a smile than to gratify scientific curiosity. Mr. W. alludes to a case of Mr. Freeke's, in which the exostoses branched like coral, projecting from the vertebræ of the neck, along the whole length of the spine, and proceeding to join corresponding productions which arose from the ribs. The luxuriant depositions of bone after injury, and the new bony sockets which attend on unreduced dislocations are glanced at by our author.

He observes that the symptoms of this form of exostosis are such as its mechanical situation or form occasion by their disturbance of contiguous parts. No remedy exists, with the exception of removal by a surgical operation, the propriety of which must be determined by circumstances and the judgment of the surgeon.

Such is Mr. Wickham's account of the periosteal exostosis. He next adverts to the medullary variety.

This, according to Sir Astley Cooper, has its origin from the medullary membrane of the bone. It is called by some osteo sarcoma, consisting of a fleshy tumor, intermixed with a deposition of bony matter. It may form in any bone, but Mr. W. believes that the maxilla, humerus, and femur, are most subject to it.

"I am much inclined to believe that this disease, although malignant in its action, yet communicates none of its specific characters to the other structures of the joint: it raises an inflammatory process on whatever texture it may exert a pressure; but the inflammation thus produced will not differ from the ordinary inflammation of the respective structures. In the cartilage it produces absorption—in the synovial membrane increased vascularity—in the cellular membrane such a deposition as is seen under inflammation caused by other circumstances, but in none the peculiar characteristics of malignant structure." 12.

Mr. W. observes that this disease is not painful in its early periods, but the pressure it produces on sensible parts renders it very much so in its subsequent stages. In its origin the diagnosis is obscure, it becomes more clear when the disease has attained some degree of magnitude. When the tumor has commenced at a little distance from the joint, it will generally have displayed its formidable character before it reaches the articulation. The constitution sympathises with this, as with all malignant diseases. No general treatment effects its cure, and operations are too commonly unsuccessful.

We stated that we doubted the strict propriety of the views of Sir Astley Cooper on periosteal and medullary exostosis. The inexperienced surgeon would be led to the conclusion, that the former is never, and the latter always of malignant character. That this conclusion is not exact will be evident, from the mention of a case which occurred at St. George's Hospital. The patient, a young woman, had a tumor in the upper part of the thigh, which had most of the characters of fungus hæmatodes. The limb was amputated immediately below the joint by Mr. Keate. The tumor proved to be of fungoid nature, and it sprung from the periosteum of the femur. In another instance, a similar tumor arose from the periosteum of the lower end of the same bone.

The fact would seem to be, that malignant growths may originate in any texture of the body, though probably the cellular constitutes their most universal nidus. The cancellous structure of the bone is certainly more disposed to their formation than the surface or the periosteum. Yet the latter is not altogether exempt, and, therefore, the doctrine that periosteal exostosis is simple, and medullary exostosis malignant in its nature, must be looked on as only generally true.

There are more varieties of exostosis than those which have been enumerated by our author, Mr. Wickham. Some appear to spring from the cancellous structure, although evincing no malignant tendency. Others arise from the periosteum, or from between it and the bone, and display a radiated character. We are not in possession of a really good account of exostoses, and the profession would feel indebted to any surgeon who would offer a lucid and complete arrangement of their numerous varieties.

Mr. Wickham passes to the subject of necrosis. His remarks are ingenious, but are not possessed of sufficient novelty to demand particular attention. We will merely advert to one practical point. It is this—that in cases of disease of the bony structures, whether that disease be caries or necrosis, Mr. Wickham has observed a disposition to frequent attacks of inflammation of the skin over the joint, of an erysipelatous character.

"This is a circumstance which I do not speak of as an established symptom of diseased bone, but I have seen it so constantly accompany it, and so seldom occurring when the other textures are alone diseased, that I lay great stress on it as indicative of a morbid state of the bony parts of the joint. I would wish it to be understood, that although the conviction on my own mind is strong as to the co-existence of this disposition to a superficial inflammation and affection of the bone of the joint, yet I am desirous only of leading others to observe for themselves. Where necrosis occurs in the continuity of the limb, I have likewise remarked the same inclination to repeated attacks of inflammation of the skin, and apparently recurring on the dislodgement of dead bone, or particular efforts in the part to get rid of the malady. When the joint is secondarily affected, in consequence of the extension of disease from the body of the bone, the existence of this complaint is self evident long before the joint becomes involved, and needs no particular hint to discover it." 20.

There can be no question that erythematous or erysipelatous inflammation of the surface, is a frequent attendant on disease of deeper parts. Thus, in cases of inflammation of the brain and its meninges, after injury of the head, it is not uncommon to observe an erysipelatous redness of the scalp. In cases of the purulent deposites in the joints, we have frequently noticed an erythematous redness of the skin, indeed we have seen the latter mistaken for rheumatism or gout. When we state these facts, the doubting reader may receive the statement of our author with less suspicion than, otherwise, he might feel disposed to extend to it.

From necrosis we slip, by an easy gradation, to caries, which occupies twenty pages of the work. Mr. Wickham is probably unused to composition, for this and other chapters are not arranged with that connected clearness which characterizes the practised writer. Mr. Wickham concludes the subject by an admission, with which we shall venture, by transposition, to commence.

"The scrofulous affection which Mr. Brodie has described as originating in the cancellous structure of bones, does not appear to me to be a distinct and separate disease from that which I have described under the heads of necrosis and caries; but that the morbid softness which he has rightly designated as scrofulous, is to be found wherever necrosis or caries exist in a scrofulous habit of body. It seems to me to be a modification of that mollities ossium which is observed in extreme instances of scrofulous disease, but in these cases confining itself merely to the articulating ends of the bones.

I have in very many instances witnessed this state of the epiphyses, the accompaniment of necrosis or caries, also where both have occurred together in the same bone. I have also dissected several joints in the same subject, in which their bones at the ends could be cut through with a knife, yet no progress had been made towards even the least diseased action.

I am inclined, then, to regard this morbid state of the bones to be that state favourable to, and often the forerunner of caries or necrosis; but not as a diseased action sui generis." 42.

Mr. Wickham appears to misconceive, in a slight degree, Mr. Brodie's views. The latter describes an affection frequently observed in persons of a scrofulous habit of body, and displaying the following series of phenomena:—First, the cancellous end of the bone is more vascular than natural; secondly, the cells are occupied by a yellowish deposite, the vascularity being diminished, and the bony matter so far absorbed that the part may readily be cut with a knife; thirdly, ulceration occurs in the morbidly-altered part, and caries or necrosis, or both, supervene.

Now, Mr. Wickham might discover a preternatural softness in the ends of many bones in the same individual, unaccompanied with any farther disease, and yet the discovery would not militate against the justice of the views of Mr. Brodie. That gentleman makes yellowness and softness of the cancelli the initiative of caries. As such, it may, and it often does exist, before caries is established. Nothing is more common than an instance of this description :—A scrofulous patient has a limb removed for caries of one of the bones of the ankle. On inspecting the articulation, the lower end of the tibia, we will say, is carious, or a part of it is dead. The contiguous portion is soft, and exhibits the yellow deposit. But the fibula and the astragalus, nay, the tarsal bones unconnected with the ankle-joint, are extremely softened also, and disclose the same yellow deposition as the tibia, although they are not attacked with caries. The experienced surgeon would scarcely doubt that the latter would have ultimately followed, had the morbid process been permitted to continue.

We recommend our readers to consult the work of Mr. Brodie, and to weigh the amount of evidence upon the subject adduced by that philosophic writer.

To revert to Mr. Wickham's description of caries. He observes that it consists of a morbid absorption of the bone, a condition analogous to ulceration of the soft parts. It is for the most part the result of inflammation and abscess of the periosteum or medullary membrane; but it is occasionally seen without the least appearance of purulent matter, and without any vestige of inflammation. This disease, as well as the forementioned affections, have their origin either within or external to the capsular ligament.

Mr. W. remarks that primary caries within the capsule is of rare occurrence, ulceration most commonly commencing in the cartilage, and spreading from that to the bone. But the caries which originates in the head of the bone, and constitutes the "scrofulous disease" of Mr. Brodie, is certainly not rare. That disease is more common in youth than in after life, and in youth, the articulating head is an epiphysis. Whether caries, commencing in the latter, is considered by Mr. W. as an instance of caries within the capsule, we feel at a loss to determine.

"Of the cases of caries which have their origin external to the capsule, and, ultimately, proceed to the implication of the joint, they are of two kinds: the one originating in inflammation of the periosteum, the other in inflammation of the medullary membrane within the bone. The periostitis at first confining itself to the membrane covering the bone, by continuance leads to the formation of matter on the outer table of the bone, to its absorption, and to the transmission of the inflammatory action to the interior, by which matter has an extensive range within the cavity and the extremities of the bones, from their greater extent of surface within, become more entirely occupied by it. At this time, diseased action having gone thus far, having had its commencement without, is on a par with disease which begins in the medullary membrane. The epiphyses become absorbed at their internal table, and a deposit takes place on the outer surface, which gives to the bone a feeling of expansion; it is this that has given rise to the term *spina ventosa*." 26.

Periostitis, therefore, may give rise to caries, as it does to necrosis, or to both. Of this all surgeons are aware. But Mr. Wickham seems to attach more importance to the form of caries which commences by inflammation of the medullary membrane, and abscess within the cancellated structure of the bone. He identifies this with the disease which has been termed the spina ventosa—" the true white swelling."

" Although I would not profess myself to be an adherent to such ancient terms, and retain these as the most appropriate to our modern nomenclature, yet I would regard them as significant of certain changes which take place in bones, and of certain states of constitution, in which this disease particularly occurs ; such as have been very long observed. The term *spina ventosa* expresses that expansion of the bone which is supposed to exist because of the enlargement which actually takes place ; and the term white swelling marks that weakness of constitution which is so evident in a scrofulous habit of body, in which this disease most frequently exists." 29.

We cannot agree with Mr. Wickham in the propriety of retaining either of these terms. The first is the expression of a pathological lie, the other is so vague as to signify nothing. If there is caries, term it caries—if necrosis, name it such—if abscess, call it abscess—but do not retain an absurd, and therefore a mischievous nomenclature, which has been a source of error and confusion. Spina *ventosa* implies, if it means anything, that the bone is inflated by aerial matter, a supposition which we need scarcely say is ridiculous. On the other hand, the term *white-swelling* cannot be said to mark that weakness of constitution so evident in a scrofulous habit of body, inasmuch as it attends affections of the joints not connected with scrofula in any way whatever.

Mr. Wickham remarks that the symptoms of caries originating above the capsule are by no means obvious. They resemble those of ulceration of the cartilage, but are more severe, and, as in necrosis, there is a great disposition to frequent attacks of erysipelatous inflammation. This circumstance, however, is not sufficiently determined to constitute a diagnostic mark on which much reliance should be placed.

" It will be found that the pain is deep in the joint, and in one determined spot ; that there is also a remote aching pain of the limb ; that these pains are much aggravated on pressure or friction of the diseased surfaces : that the pain of the joint is of the pricking character, and as if some animal was eating the bone away.

These symptoms are at first unaccompanied by swelling, which only occurs as the other structures may be effected : neither is there at first deformity of the joint, which only appears as a subsequent symptom on the destruction of much of the bony part of the articulation, or under continued muscular action, after the ligaments have become loosened from their natural holds." 33.

The symptoms of caries extending from the body of the bone to the joint, are so manifest as not to require description.

The characters of medullary inflammation and spina ventosa are not so readily understood at first, from the depth of pain, and the want of any external mark of disease in the commencement. Mr. W. relates four cases of this variety of caries. The following is a notice of the morbid changes in an elbow-joint affected with the malady. The extremity of the ulna, the olecranon especially, was greatly enlarged, and the interior of the bones at these parts much excavated, as if worm-eaten, the cartilages were quite destroyed, and the other parts much altered ; the condyles of the humerus were involved in the same disease.

Mr. Wickham gives an instance of the combination of caries with necrosis. It forms a fair illustration of what has been denominated spina ventosa, prior to destruction of the joint.

*Case.* The patient, aged 18, was admitted into the hospital, November 30th, 1831. Two months previous to his admission an abscess formed anteriorly on the upper part of the tibia, and having discharged a considerable quantity of matter, eventually healed. The bone, at the time of his admission into the Hospital, was enlarged for almost one-third of its upper part. The integuments covering the part were discoloured, and in an unhealthy condition. Successive blisters were directed to be applied over the seat of disease; the constitution, which was debilitated, by previous illness, being supported by bark and a generous diet. This treatment was pursued for six weeks, when the patient began to complain of an aching pain in the knee, which was much increased by pressure under the patella. The tumefaction of the knee was evidently increased, and the head of the tibia somewhat more enlarged. An issue was ordered in front of the tibia, two inches below the knee-joint. This produced a very beneficial result, and the inflammation which had threatened the joint was completely arrested. Three weeks afterwards the leg was attacked with erysipelas, which was so severe as to cause the death of the boy.

On inspection of the joint, the marks of incipient inflammation were present on the synovial membrane, the other parts of the joint being in a healthy condition. The head of the tibia was much enlarged, very rough, and the inner surface carious, the ulcerative process having thinned the walls of the epiphysis considerably. The cancellated structure was necrosed.

The treatment of caries should consist of local abstraction of blood, by means of leeches, in the first instance, and the employment of the caustic issue afterwards. Perfect rest must be enjoined, and maintained by mechanical means, such as splints, a convenient angle and position being chosen. Occasional leeches and cold applications are not unfrequently required for the repeated attacks of superficial inflammation.

"If it is found that an outlet can be given to the small carious fragments, or to purulent matter within the bone, incision or the trephine should be applied at that part which seems to offer as the most eligible. In these cases it is desirable to remove the whole carious surface to induce new granulations from the sound bone, not to rest satisfied with simply dislodging the purulent matter, or the necrosed bone." 41.

This is an important piece of advice, and one which should be treasured in the minds of surgeons. Limbs have been lost, from the neglect of the trephine. The instances of abscess in the cancellous ends of the tibia, related by Mr. Brodie in the last volume but one of the Transactions of the Medico-Chirurgical Society, are remarkably in point. In cases of necrosis of the tibia the trephine is very useful. In short, if the surgeon suspects the confinement of matter in a bone, or dead bone locked up and proving a source of irritation, it is his duty, if the parts are accessible to an operation, to set the offending substance free. The trephine is to the hard parts what the lancet or the scalpel proves to the soft, and should be used, with reservation, upon similar principles.

When we cast a retrospective glance at the chapter we now quit, we perceive that Mr. Wickham has succeeded in directing the attention of the reader to the characters and varieties of caries. It is an affection deserving of consideration and attention, and one which is probably far from exhausted.

But we feel some hesitation in admitting what the reasonings of our author seem intended to imply, that the spina ventosa, such as he describes it, constitutes the ordinary strumous disease of the articulations, or, in other words, "the true white-swelling." Independently of the consideration that the idea is not borne out by a sufficient number of well-recorded cases, we cannot shut our eyes to the evidence contained in the works of Mr. Brodie, and the facts that Nature constantly presents. It is true that caries of the osseous structure is more universally and strictly scrofulous than disease of any of the other tissues that enter into the constitution of the joints. But the question that occurs is—whether that strumous disease of the bone be commonly such as Mr. Wickham or such as Mr. Brodie has described. With the utmost respect for the former gentleman, we adhere to the opinions of the latter. It is not from mere deference to authority that we do so, but because the many cases we have witnessed in a large hospital have been perfectly confirmatory of the views of the able surgeon we refer to.

Our readers will observe that caries of the articulating ends of bones may occur under several and different circumstances. It may result from the extension of inflammatory action from the cartilages or the other tissues of the joint—it may originate in the cancellous structure of the bone, as a sequence of ordinary inflammation of that structure—it may occur in the manner described by Mr. Brodie, under the denomination of scrofulous disease of the articulation—it may follow periostitis or disease of the bony shaft. They will also remark that, under any of these circumstances, the caries or ulceration may be complicated with necrosis, or more or less extensive death of the bone—that abscess may coexist in the joint, or the soft parts—and that, in some cases, an abscess may form in the cancellous tissue alone, independently of any affection of the joint. It is especially in the latter instance that the use of the trephine is demanded.

The profession do not appear to be generally aware how serviceable the trephine is in cases of necrosis. If the surgeon waits till the sequestrum comes away *sponte suâ*, or till Nature has established such outlets that it may do so, the case is protracted to an unnecessary duration. But if he anticipates the natural process, by trephining the new bone as soon as it has acquired sufficient strength to support the limb in an adequate degree, and by removing the sequestrum through the artificial opening, he saves the patient much time, much suffering, and much injury to the powers of the constitution.

Mr. Wickham proceeds to diseases of the cartilage, and from them to inflammation, acute and chronic, of the synovial membrane. The researches of Mr. Brodie have left so little to be done upon these heads, that we cannot be surprised at our author doing little. Three incidental points are all that we shall notice.

Mr. W. remarks that ulceration of the cartilage occurs more frequently in the hip than in any other joint. We doubt the correctness of this observation. We should feel inclined to consider the knee-joint as that most frequently attacked.

He observes that chronic disease of the synovial membrane is very common in the lower joints of horses, that have been subjected to constant battering on hard roads.

The remaining point of the three to which we alluded is connected with the influence of mercury upon the system.

"It has been remarked," says Mr. Wickham, "that the action of mercury on the constitution leads to the production of this affection. How far it is the specific change affected by this remedy on the membranous structure, or that in common with other depressing causes it induces a condition of the system particularly favourable to the production of synovial inflammation, I cannot decide. It renders the system peculiarly susceptible to the impression of cold, and cold is one of the principal causes of this attack." 80.

Inflammation of the synovial membrane occurs under two sets of circumstances—in combination with venereal symptoms requiring mercury, and with those cachectic symptoms too frequently brought on by the abuse of that mineral, and contra-indicating its employment. The latter is the more frequent, and the surgeon would commit a lamentable error who should put the patient under the influence of mercury, for synovial inflammation of this low character.

It is rather singular that Mr. Wickham makes no mention of calomel and opium nor of colchicum in the treatment of acute synovial inflammation. They are both, to say the least, important remedies. Neither does he draw attention, at least not distinctly, to the secondary inflammations of the membrane which follow injuries and operations.

Mr. Wickham devotes fourteen pages to the consideration of disease of the cellular membrane of the joints. Some of our readers may remember that a few years ago we reviewed a paper on this subject from the pen of a French surgeon. The object of that writer was to prove, that the affections of the cartilage and bone most commonly originate in the cellular tissue external to the articulation. In that he certainly failed. Inflammation and its consequences frequently invade the cellular tissue, as a consequence of disease of the deeper parts, but although the reverse occasionally happens, we have no precise data for its frequency, nor any unexceptionable account of the process.

Mr. Wickham divides the inflammation of the cellular tissue of the joint into two varieties;—that which attacks the cellular substance within the joint, and that which occupies the cellular substance that invests the capsule. In the former case the swelling is but slight, in the latter it is considerable, and the disease is generally more active in its progress.

"The first stages of inflammation of the cellular membrane, whether superficial or deep-seated, whether circumscribed or having an extended range around the joint, are nearly the same, differing only in degree. The swelling of the joint is equable and firm, and as the skin becomes placed on the stretch, becomes shining and white; this stage, which may be called the adhesive, will last sometimes for many months, and produce very great irritation on the general system. As the skin becomes placed on the stretch to a great degree, its sensibility is much quickened, and the sufferings of the patient consequently much aggravated, the person not being able, at times, to bear the weight of the bed-clothes. I have been entreated, on this account, to make an incision of some length, with a view to relieve the tension and pain which accompany it. The pain, until the arrival of this symptom, is more obtuse, and oftentimes but very slight.

The duration of this stage varies very much, but the approach of that which is attended by the suppurative process, is for the most part marked by the usual constitutional symptoms, indicating the formation of matter; such as rigors, and succeeding heat and general febrile paroxysms, which assume, if the suppuration be large, an hectic character. It sometimes happens that the constitutional disturbance is so great as to require the removal of the limb, and that before any other structure is implicated; but it is more common for inflammation to propagate itself by contiguity to the synovial membrane, and afterwards to the articulating cartilages, when it becomes confused with the peculiar symptoms attendant on disease of those parts.

Two kinds of cases are presented to us by disease of the cellular membrane; the one in which a single or more spots may have been the seat of the inflammation, having its origin from some injury which the part may have received, and pursuing a chronic course to the formation of small sacs of pus in those situations, which perhaps ulcerate through the synovial membrane. The second case is that in which the whole of the cellular membrane surrounding the articulation becomes inflamed, and ultimately envelopes the joint in one large abscess. The first case is the more common of the two; the latter the effect of a sudden attack of inflammation, and more active in its course." 86.

Mr. Wickham relates some cases illustrative of his descriptions. The following is an instance of the first form of cellular disease.

*Case.* Maria Pratt, admitted into the Winchester Hospital, Jan. 9th, 1822. The left knee-joint was greatly enlarged, hard and elastic to the touch, the skin over it very tense and shining, and (with the exception of marks which had been produced by issues and other remedies) of a whitish hue. Her pain, at the time of her entrance to the hospital, was very severe, and had been so for a considerable time. It was deep on each side of the patella; her idea was that she could cover each place with a shilling-piece. She had suffered from the disease for two years, and anxiously submitted to amputation of the limb. Dissection of the joint shewed the cellular texture and parts external to the joint, indurated and converted into a firm jelly-like mass, having somewhat the appearance of the horn of brawn. Three sacculi of matter were found in the front of the joint, two on one side, and one on the other side of the patella (the original seat of pain); one sac was on the point of ulcerating through the synovial membrane. That texture appeared perfectly healthy, and the cartilages and bones were without any change from their natural state.

In another case, in which amputation was performed by Mr. Wickham's father, examination of the limb disclosed these particulars. The knee was immensely swollen by a firm and hard mass, under which the sac of an abscess was found, passing nearly half way up the thigh, along the rectus muscle; the whole circumference of the joint was also occupied by this sac. The discharge had been very copious. The synovial fluid was, by the irritation of matter external to the capsule, somewhat increased, but the vascularity of the membrane was not apparent, neither did any disease shew itself on that structure or any other part of the joint.

In a case in which the disease was observed as it passed through all its stages, dissection displayed its formidable ravages. The cellular substance was about an inch thick, firm and jelly-like; an abscess on the fore part, and on each side of the joint, of very considerable size, and communicating with the cavity by an ulcerated opening at the upper part; the synovial membrane was vascular and flocculent, the cartilaginous surfaces in several parts ulcerated, and that on the inner side of the patella laying bare the bone, which was somewhat carious. The direct ligaments appeared alone to be uninflamed.

The causes of the complaint are stated by Mr. W. to be injuries, or the application of cold.

The treatment is obvious:—local depletion for the early stage of the complaint—rest for all. Friction has been found by our author prejudicial, counter-irritants have been rather productive of mischief than advantage. But he speaks highly of Mr. Scott's plan of moderate pressure, combined with the application of mercurial and camphor ointment.

The preceding remarks are interesting and important, being calculated to direct attention to a form of disease, which, though not so frequent as affections of the proper tissues of the joints, is still of occasional occurrence, and must not be forgotten nor neglected. It must be owned that the symptoms laid down by Mr. Wickham are not sufficiently distinctive to preclude all risk of misconception. The equable and firm swelling of the joint, and the glossy and white appearance of the skin, are characters not unfrequently observed in cases of ulceration of the cartilage. Probably the main diagnostic features would be found in the pain on motion or on handling of the surface, unaccompanied with a corresponding degree of suffering on pressure of the articulating surface of the tibia and the femur, or of both those bones and the patella. The latter is a prominent symptom of ulceration of the articular cartilage, with which the chronic cellular disease is most apt to be confounded. We have at the present moment a lady under our care, in whom the history of the disease leaves little doubt that it commenced with inflammation in the cellular tissue; at present there is, unfortunately, ulceration of the cartilage with abscess of the joint.

Acute inflammation and diffused suppuration in the cellular membrane external to the joint, is not uncommon as a consequence of cold in persons of debilitated constitution. We have seen many fatal cases of this nature. The joint became affected in all. Our space will not permit us to speak more at large on this affection here, but perhaps we may return to it at a fitting opportunity, and lay some remarkable cases before our readers. In conclusion, we beg again to draw attention to the observations of Mr. Wickham.

Diseases of the ligaments next occupy the attention of our author. Like most practical surgeons he has come to the conclusion, that though they may be liable to primary disease, they are usually found to be the last of the articular tissues which suffer. We extract a few notes on relaxation of the ligaments.

> "Boys are frequently brought to the Hospital, from the country, who have followed the plough with boots of an immense weight. They have lost the arch of the foot, and the astragalus falls to the ground, protruding inwards. At the same time the muscles of the leg (the gastrocnemii) are wasted. These boys walk principally by the muscles of the thigh, dragging their leg, rather than lifting it, in progression.
>
> We see frequently, also, the knees of young persons turning inwards by a lateral inclination, the internal lateral ligaments yielding to an irregular and unnatural pressure. From this cause I have seen a young man whose left leg was so affected by this lateral inclination, that the leg was nearly at right angles with the thigh. Yet, with this extraordinary deformity, no disease existed." 100.

The treatment can only consist in avoiding injurious practices or habits—

in obviating the influence of weight on the weak part, and in giving it a different direction—and in imparting general health and vigour.

Our author proceeds to the general terminations of diseases of the joints —abscess—anchylosis—and constitutional implication.

Mr. Wickham observes that abscesses not uncommonly form in the muscles at a considerable distance from the joint, and that when the original disease in the latter shall long have been quiescent. Their magnitude is often very considerable where they form among the large muscles in the neighbourhood of the hip and knee. Mr. W. has known an abscess amongst the extensor muscles of the thigh in a case of diseased knee, reach to within three inches of the hip-joint.

"It may be observed that such collections of matter most frequently happen amongst the extensor muscles, which move the lower bones of the affected joint. This is a fact which to my knowledge has not been noticed, but it seems to me to deserve attention, inasmuch as it explains the cause of dislocation which happens by the forcible contraction of the flexors, the power of the extensors being destroyed. In the disease of the hip joint, the glutei are for the most part the seat of abscess. When the knee is affected, abscesses generally form in the bodies of the rectus femoris and vasti. When the elbow is diseased, matter forms in the triceps extensor cubiti. These collections of matter do not occur so frequently in the neighbourhood of the smaller joints, which are surrounded by tendons, rather than the bellies of muscles, and have the cellular substance more condensed.

It is the destruction of the extensor muscles by inflammation and abscess, that leads to the dislocation which occasionally happens by the contraction of their antagonists. The power of one set of muscles being destroyed, the action of those opposed to them is thereby induced, and rendered involuntary and permanent." 106.

In speaking of anchylosis, which, our readers are aware, may result from three causes—muscular contraction—effusion of lymph—and deposition of ossific matter, Mr. Wickham adverts in a forcible manner to the former. If one class of muscles, the extensors, become wasted in the progress of the malady, their antagonists will act and of course will tend to displace and to dislocate the limb.

"In the progress of disease of the shoulder, the deltoid becomes wasted, and anchylosis, with a *partial dislocation*, is produced by the powerful action of the pectoralis and latissimus dorsi acting in concert. In cases in which the elbow is the subject of disease, the triceps extensor cubiti is found wasted and gone, and the biceps shortened and rigid, the arm becoming anchylosed at right angles. In extreme cases the condyles of the humerus are seen to project, giving the appearance of a partial dislocation of the radius and ulna forwards. After disease of the wrist, the hand will be permanently flexed, turned with the palm upwards towards the fore part of the arm. In the fingers every one will have observed the rigid contraction of the flexor tendon when any joint has been diseased and become stiffened. In the ankle a semiflexion is performed, the sole of the foot turning inwards." 112.

It is the duty of the surgeon to be acquainted with this circumstance—it should be his object to prevent it by suitable position and mechanical contrivance.

The principles of treatment of anchylosis laid down by Mr. Wickham are judicious. We fear, however, that he leans too much to friction and to force.

Our author occupies two or three pages with the question of amputation. The concluding forty pages of the work are dedicated to the description of disease of the hip-joint. The chief points of interest connected with this disease are the apparent elongation of the limb in its commencement, and the shortening at its conclusion.

The first has been proved to be deceptive in its character. The general opinion on its nature is this:—that the pain on motion and progression induces the patient to relieve the affected joint by bearing his weight on the unaffected limb, that this, after a time, gives rise to lateral curvation of the spine, which establishes and increases the apparent deformity.

"The opinion generally entertained at this present time is, that the appearance of elongation arises from the distortion of the spine, by a descent of the pelvis on the affected side, in consequence of the patient throwing the weight of his body on the opposite limb—an effect rather, as it is said, produced by the habit which the person contracts in walking than by the existence of disease of the spine in connexion with that in the hip-joint." 148.

We doubt whether the majority of surgeons entertain this opinion, to the extent expressed in the preceding passage. The opinion in question has rather reference to an advanced, than a *very* early state: for, as position produces the distortion of the spine, and is not altered, but only maintained, by that distortion, it follows as a deduction, both of reason and of logic, that at first the apparent elongation of the limb is independent of actual curvation of the spine. However this may be, Mr. Wickham's explanation of the circumstance is ingenious, and probably in many instances, correct. It is this. He starts by admitting that, in some cases, there is lateral curvature of the spine, and alteration of the horizontal plane of the pelvis, but he denies that this is the usual condition.

"The deception is, I conceive, produced by the position into which the limb is thrown by rigid muscular action. It will be observed that the leg is projected away from its fellow, and the toe is turned a little outwards, so that a person comparing the two legs will bring the sound towards the affected, rather than by great force and consequent pain, draw the inflamed limb towards the sound one. Thus the sound leg does not traverse the median line without losing from an inch and a half to two inches of its length. If this be admitted as a sufficient explanation of the deception as to the actual lengthening of the diseased leg, our next inquiry will be to ascertain the cause of the rigid muscular action which projects the foot so far away from its fellow and leads to the appearance.

The muscles which extend the thigh upon the pelvis, which carry the thigh backwards and outwards away from the opposite limb, are the glutei, aided by the rotators, which act in concert with them.

*To the inordinate and powerful action of these muscles*, then, I attribute the projection of the limb outwards. That they are capable of this effect, their ordinary action clearly points out. That they do produce this effect appears to me reasonable, from the following circumstances. Towards the termination of the first stage, and throughout the after periods of diseased hip, we find the glutei muscles undergoing some change; the first observable alteration is their wasting; the second, perhaps, is the formation of matter in their substance, and their total obliteration.

Thus we see these muscles engaged and involved in the progressive stages of diseased hip, by very palpable and evident alteration of structure. It may be hence argued, and it is to be supposed, if not beyond doubt, that some active state has preceded that of wasting; and it is to me equally evident, that the inordinate action of the glutei which throws the limb outwards, is that state which precedes the wasting of these muscles. I consider, then, that the glutei muscles, from their contiguity to the inflamed joint, become irritated to excessive action, which projects the leg outwards, and that this action is the cause to which the apparent lengthening of the leg may be fairly and entirely attributed." 159.

The question, then, is before the profession—whether the apparent elongation of the limb is due to inordinate action of the glutæi and rotators—or to position (implying the associated action of more muscles than the preceding, but including their contraction), and, subsequently, to actual distortion of the spine. Mr. Wickham's ingenious supposition is highly deserving of consideration.

In the second stage of disease of the hip-joint, there is shortening of the limb affected. Mr. Wickham remarks that this may be *apparent*, and without dislocation of the head of the femur from its socket: or *real*, and dependent upon such an alteration.

" I am the more anxious to dwell on this distinction, from the belief that the subject has not received the consideration of the profession, and from the circumstance that I have repeatedly seen cases pronounced to be in a state of incurable dislocation, which have proved otherwise; and that, too, by surgeons for whose talents and judgment I entertain the highest respect. According to my own experience, the actual dislocation of the thigh is of very rare occurrence. I do not think I have met with one case in twenty of the shortened leg in which I could pronounce the bone to have escaped from its socket." 162.

We believe it is admitted that shortening may exist independently of dislocation, from ulceration of the head, and partial or general destruction of the cervix of the femur. Such an admission is contained in the work of Mr. Brodie.

" In the very advanced stage of the disease (says he) when the head of the femur has been completely destroyed by ulceration, there is nothing to prevent the muscles from pulling the bone upwards. This may be compared to a case of fractured neck of the femur. The limb is not only apparently, but it is really shortened : the foot may be rotated inwards, but, if left to itself, it generally is turned outwards."—*P.* 161, 2*d Ed.*

Mr. Wickham proceeds to the pathology of the stage of *apparent* shortening. We confess we have misgivings of the propriety of the designation. Take, for instance, the following cases, intended, we presume, to illustrate his observation.

*Case.* Mr. W. dissected the hip-joint of a man, who died under long-protracted disease of the articulation. The limb appeared much shortened, and drawn over its fellow. On exposure of the parts, a confused mass presented itself of jelly-like matter, mixed with dark pus, having small fragments of bone floating in it; no vestige of the natural textures of the joint remained ; the head of the femur was almost destroyed by absorption, a portion only remaining about the size of a walnut; it was imbedded in the acetabulum, which was divided into three portions, and so thin as to break down under the finger ; matter escaped into the pelvis, behind the peritoneum.

Surely there must have been real shortening here, the head of the femur being almost gone, and the cavity of the acetabulum deepened by ulceration.

*Case.* In the case of a female, in whom the limb was thrown over its fellow, and more drawn up towards the abdomen than Mr. W. had ever previously seen it, the soft parts of the joint were found entirely destroyed, and a dark offensive matter ranging in the cavity which was once the articulation; the round ligament and cartilage were gone from the head of the femur and acetabulum; the upper and outer edge of the socket was nearly absorbed, on a line with the surface of the dorsum ilii, and the head of the bone lay imbedded in the excavation.

In another case the affected limb was drawn upwards and forwards, so as to present the appearance of shortening to a considerable degree; the ball of the great toe reached about four inches above the ankle of the opposite leg, and the thigh crossed the other. On examination, the head of the bone is described as remaining within the acetabulum, and matter, which had formed in connexion with the hip-joint, communicated with the rectum. But no more particular account is offered.

It cannot escape observation, that Mr. Wickham has made a very serious omission in these cases. He has neglected to ascertain, at all events to mention, whether the shortening was ascertained to be real or deceptive by actual measurement in the living patient. In all such cases, it is the practice of the careful surgeon to learn, by the application of tape, whether there is actual shortening of the limb, or whether the apparent diminution of length is due to position, or to alteration of the pelvic plane. The omission to which we have alluded is equally conspicuous in some cases that Mr. Wickham next relates, in which the shortening disappeared under proper and efficient treatment. The following, as the strongest, is perhaps the best instance.

"————, in July, 1831, was admitted into the Hospital, weakened by the continuance of disease of the hip-joint. He was placed at once in bed, and not submitted to the usual forms of the house, so weak was his condition. His limb was much distorted and bent on the body, and he lay on his right side, his disease being in the left hip. Actual disease had only existed a few months, but he was reduced to his debilitated state by an attack of scarlet fever. His pain at the joint and at a distance was very great.

A seton was applied in the groin, and another opposite the trochanter. These afforded considerable relief. He was kept in bed, and a liberal diet allowed him. Under treatment the diseased action entirely subsided after three months, and he quitted the hospital, with his foot reaching to the ground the limb only weak.

This case was seen by an eminent surgeon of one of the large hospitals in London, and pronounced to be in a state of actual dislocation from disease. This opinion I combated at the time, with, I hope, that deference which was due to his great and distinguished talents, but at the same time with that confidence which my own peculiar views and extended observations inspired me with." 169.

Here we have no means of discovering whether the shortening was genuine or fictitious, whether the eminent surgeon of London was very negligent, or slightly culpable. At the same time, we give much credit to Mr. Wickham for prominently directing attention to the fact, that great alterations in the appearance of the limb, and position closely resembling that of dislocation, very frequently exist, independent of that change. There can be no question that routine surgeons, of whom there is no lack, even among those attached to hospitals, are frequently deceivers and deceived, from ignorance or inattention.

The only point to which it remains for us to allude, is the manner in which dislocation occurs. It is commonly attributed to the action of the glutæi, but such is not the opinion of our author. In his view of the alteration, the iliacus and psoas play the chief parts.

"When enumerating (says Mr. W) the symptoms of the first stage of the diseased hip, I alluded to the flatness of the nates, produced by wasting of the glutei muscles, and spoke of it as a circumstance not only the precursor, but as the actual cause of the second stage. Whoever will take the trouble to examine into the action of the large muscles which move the femur, and observe by their insertions how the upper and lower ends of the bone must be differently affected, will be enabled to comprehend how, in the state of shortening by position, as in the state above described, the flexor muscles bring the shaft and lower part of the bone upwards and inwards across its fellow, whilst the head of the bone remains stationary in its socket, its principal pressure bearing against the outer and posterior edge of that cup, and it will be readily understood how the head of the bone will be retained in its socket until this edge be entirely absorbed, or the head of the femur itself be so destroyed as to be incapable of resistance, and thence liable to be forced backwards on the dorsum ilii. In the stage of shortening, the flexor muscles, the psoas and iliacus, are called into such action as to bring the thigh upwards and inwards in the position which has been noticed; they are called into exertion in consequence of the inaction from loss of power by wasting of their antagonists, the extensors, (the glutei)." 172.

He remarks that dislocation of the head of the bone is rare, and can only happen when the ring of the acetabulum is broken down by absorption, or the head of the femur so lessened as to allow of a wider range to its movements in the socket, by which a slight degree of irregular action may displace it. He thinks that, even in the latter case, dislocation is unusual, for the wasted head is rather disposed to be drawn into the depth of the acetabulum.

" In all cases of actual dislocation which I have witnessed, I have found the edge of the acetabulum, at its upper and outer part, entirely absorbed, and the head of the femur more or less destroyed, and lying between the acetabulum and the ischiatic notch, but rather higher on the dorsum ilii than the level of that notch." 173.

It will be seen that the description of the symptoms, that is, of the appearances of dislocation, does not tally with what is usually offered and received. It is commonly believed that the physical features of the dislocation on the dorsum ilii, from disease, agree with those of the same dislocation from accidental violence. But Mr. Wickham says that they resemble those of fracture of the neck of the thigh-bone.

" By the dislocation of the femur the length of the limb is actually shortened to the amount of nearly four inches; it loses its former position of flexion on the body; instead of being drawn forwards and inwards on the other thigh, it becomes straightened, and the knee which was turned inwards is now directed outwards, or, as is most commonly the case, moveable from the straight position to the eversion, but incapable of turning inwards. It assumes the same aspect as the fracture of the neck of the femur." 173.

It has certainly appeared to us that the ordinary notion is correct. But Mr. Wickham's statements will induce us, and probably others also, to look at the subject with more minute attention.

Here we close the volume, and part from Mr. Wickham. We leave him with feelings of esteem, and beg to compliment him on his industry, ingenuity, and zeal. If he perseveres, and we do not doubt that he will, the sphere in which he moves will acknowledge his merits and reward his exertions in a manner more significant and more substantial than mere eulogy.

